# Generation of knock-in lampreys by CRISPR-Cas9-mediated genome engineering

**DOI:** 10.1038/s41598-021-99338-1

**Published:** 2021-10-06

**Authors:** Daichi G. Suzuki, Hiroshi Wada, Shin-ichi Higashijima

**Affiliations:** 1 Exploratory Research Center On Life and Living Systems (ExCELLS), National Institutes of Natural Sciences, 5-1 Higashiyama, Myodaiji-cho, Okazaki, Aichi 444-8787 Japan; 2 Faculty of Life and Environmental Sciences, University of Tsukuba, Tsukuba, 305-8572 Japan; 3 National Institute for Basic Biology, National Institutes of Natural Sciences, 5-1 Higashiyama, Myodaiji-cho, Okazaki, Aichi 444-8787 Japan

**Keywords:** Genetic engineering, Evolution

## Abstract

The lamprey represents the oldest group of living vertebrates and has been a key organism in various research fields such as evolutionary developmental biology and neuroscience. However, no knock-in technique for this animal has been established yet, preventing application of advanced genetic techniques. Here, we report efficient generation of F_0_ knock-in lampreys by CRISPR-Cas9-mediated genome editing. A donor plasmid containing a heat-shock promoter was co-injected with a short guide RNA (sgRNA) for genome digestion, a sgRNA for donor plasmid digestion, and Cas9 mRNA. Targeting different genetic loci, we succeeded in generating knock-in lampreys expressing photoconvertible protein Dendra2 as well as those expressing EGFP. With its simplicity, design flexibility, and high efficiency, we propose that the present method has great versatility for various experimental uses in lamprey research and that it can also be applied to other “non-model” organisms.

## Introduction

The lamprey belongs to the basal-most group of vertebrates, the cyclostomes, and retains ancestral characteristics, such as lack of a jaw and paired fins. Consequently, this animal has attracted attention from a wide range of researchers, including evolutionary developmental biologists and neuroscientists^1–6^. However, its long, complex life cycle^7^ prevents establishment of genetic lines, and thus it had been practically impossible to apply modern genetic techniques to the lamprey until quite recently. Now, F_0_ knock-out lamprey mutants can be generated effectively using the CRISPR-Cas9 system for the sea lamprey *Petromyzon marinus*^8^, Northeast Chinese lamprey *Lethenteron mori*^9^, and Arctic/Japanese lamprey *L. camtschaticum*^10^, allowing loss-of-function analysis in these species. However, many techniques, including in vivo imaging, photoconversion, calcium imaging, and optogenetics, require a knock-in method. Although I-SceI meganuclease-mediated tissue-specific transient transgenesis has been reported, its transgene integration into the lamprey genome was not either specifically targeted or determined^11,12^. In this study, we succeeded in generating F_0_ knock-in lampreys (for the Arctic/Japanese lamprey *L. camtschaticum*) expressing reporter genes, the genomic integration of which was determined by PCR and sequencing. This method is simple and versatile for various experimental aims. In addition, the efficiency of obtaining transgenic lampreys is very high (20–35% of survivors). Based on these results, we believe that this CRISPR-Cas9-mediated versatile knock-in method opens up new research horizons using lamprey and possibly other “non-model” organisms in the sense of molecular genetics.

## Results

### Strategy for generating knock-in lampreys with the *LcHsp70A* promoter

**Figure 1:**
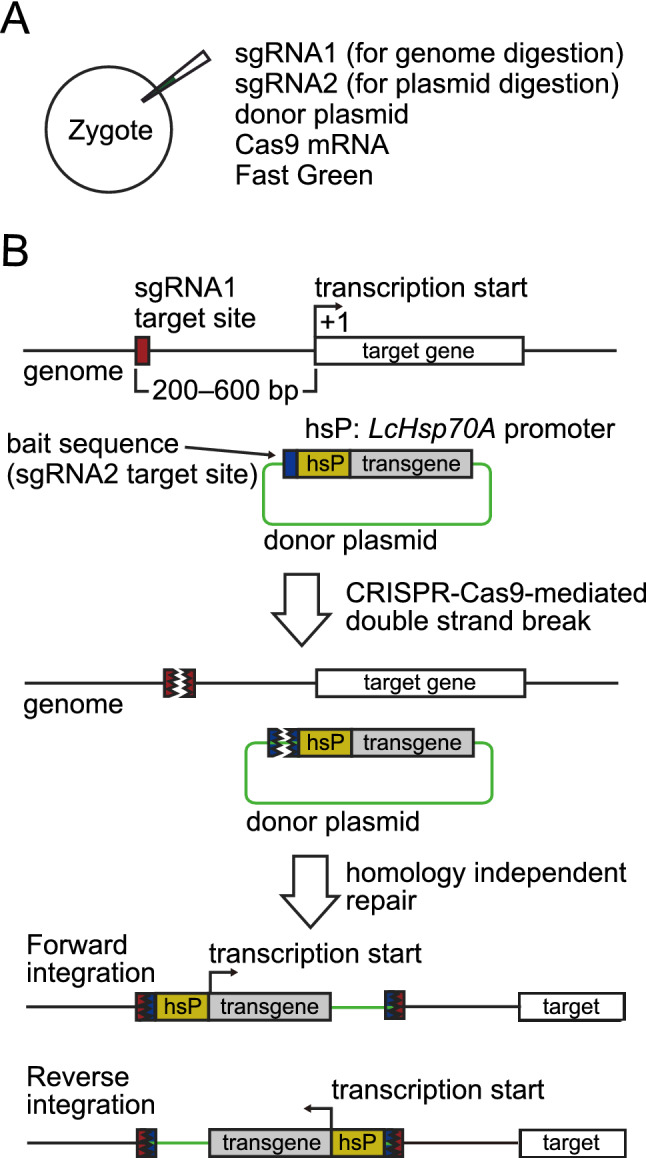
CRISPR-Cas9-mediated knock-in strategy using *LcHsp70A* promoter. (**A**) For the generation of knock-in lamprey, sgRNA1 (for genome digestion), sgRNA2 (for plasmid digestion), the donor plasmids having a bait sequence, and Cas9 mRNA in distilled water containing Fast Green to aid visualization of the spread of the injection are co-injected into lamprey zygotes. (**B**) After injection, the CRISPR-Cas9-mediated concurrent cleavage occurs in the genome at the site upstream (approximately, 200–600 bp) of the target gene and in the donor plasmid at the bait sequence. This leads to a homology independent DNA repair, resulting in the integration of the donor plasmid into the targeted locus. Here, both forward and reverse integrations can occur. Cis-regulatory DNA sequences for the target gene expression act on the *LcHsp70A* promoter (enhancer-trapping), resulting in the expression of the reporter gene in cells that express the target gene.

For CRISPR-Cas9-mediated knock-in via non-homologous end joining (NHEJ) in the lamprey, we used an experimental strategy previously established in zebrafish^13^ and medaka^14^ with minor modification: co-injection of sgRNA1 (for genome digestion), sgRNA2 (for plasmid digestion), donor plasmids, Cas9 mRNA, and fast green (for visualization of the injection cocktail) (Fig. 1A).

Species-specific *heat shock protein 70* (*Hsp70*) promoters work effectively as a minimal promoter in zebrafish and medaka^13,14^. Thus, we first aimed to isolate a lamprey *Hsp70*-like promoter. We found two homologs of *Hsp70*-like genes in the *P. marinus* and *L. camtschaticum* genomes, one of which we named *LcHsp70A*. Phylogenetic analysis suggests that those two homologs are the result of lineage-specific duplication in the lamprey (Fig. S1A). The *LcHsp70A* sequence was also found in the transcriptomes of *L. camtschaticum* embryos obtained previously^15^. In this study, we extracted a ~ 0.2 kb sequence of the *LcHsp70A* promoter for plasmid construction (Fig. S1B).

The donor plasmid contains a bait sequence (Tbait, a 23 bp sequence derived from the mouse *Tet1* gene^13^, upstream of the insertion cassette for sgRNA2-guided DNA cleavage (Fig. 1B). This bait sequence was selected because the corresponding sgRNA (sgT) appears to have no potential off-target binding sites except for those with three or more mismatches in the *L. camtschaticum* genome (Table S1). Tbait is followed by the *LcHsp70A* promoter, which we expect to work as a minimal promoter (Fig. 1B). Finally, the *LcHsp70A* promoter is followed by reporter genes (in this study, EGFP or Dendra2).

We set the target site for genome digestion approximately 200–600 bp upstream of the predicted transcriptional-start site of the target genes (Fig. 1B; the sequences used are shown in Table S2). Concurrent digestion of the genome and plasmid DNA (guided by sgRNA1 and sgRNA2, respectively) with Cas9 would result in the integration of the donor plasmid into the genome via NHEJ (Fig. 1B). Then, cis-regulatory sequences for tissue-specific expression of the target gene would act on the *LcHsp70A* promoter, resulting in expression of a transgene in cells that express the target gene (enhancer-trapping).

### Generation of *Bra*:EGFP and *MA2*:EGFP knock-in lampreys

**Figure 2:**
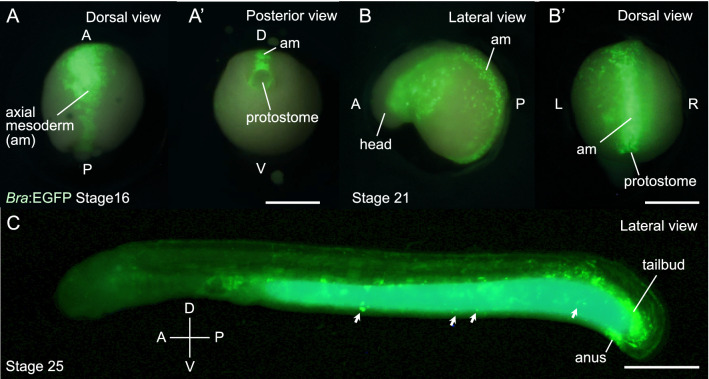
*Bra*:EGFP knock-in lampreys generated by microinjection of *Bra*-sg1. (**A**) At stage 16, in dorsal view (**A**) and posterior view (**A**’). EGFP is expressed in the axial mesodermal cells (am). A, D, P, V indicate anterior, dorsal, posterior, and ventral, respectively. Scale bar: 500 μm. (**B**) At stage 21, in lateral view (**B**) and dorsal view (**B**’). EGFP is persistently expressed in the axial mesodermal cells (am). L, P, R, V indicate left, posterior, right, and ventral, respectively. Scale bar: 500 μm. (**C**) At stage 25, in lateral view. The EGFP expression was restricted mostly in the tailbud and anal regions. In the york, some non-specific signals (by unintegrated plasmids) were observed (arrows). A, D, P, V indicate anterior, dorsal, posterior, and ventral, respectively. Scale bar: 500 μm.

**Figure 3:**
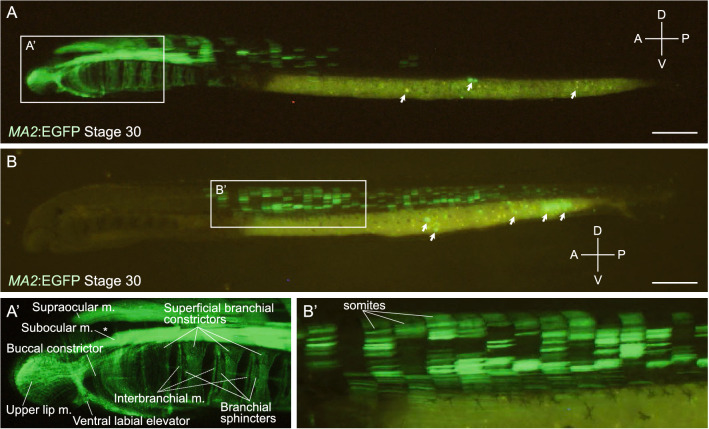
*MA2*:EGFP knock-in lampreys. (**A**) A *MA2*:EGFP knock-in lamprey showing EGFP expression in the head region at stage 30, in lateral view. The head region is magnified in (**A**’). The asterisk (*) indicates the eyeball. EGFP is expressed both somatic and pharyngeal muscles (m.). In the york, some non-specific signals (by unintegrated plasmids) were observed (arrows). A, D, P, V indicate anterior, dorsal, posterior, and ventral, respectively. Scale bar: 500 μm. (**B**) A *MA2*:EGFP knock-in lamprey showing EGFP expression in the trunk region at stage 30, in lateral view. A part of the trunk region is magnified in (**B**’). Both EGFP-positive and EGFP-negative somatic muscle cells are observed in the same somite. In the york, some non-specific signals (by unintegrated plasmids) were observed (arrows). A, D, P, V indicate anterior, dorsal, posterior, and ventral, respectively. Scale bar: 500 μm.

To examine the effect of the CRISPR-Cas9-mediated knock-in system, we first targeted *brachyury* (*Bra*) and *muscle actin 2* (*MA2*) genes for an EGFP reporter assay (hereafter, we call the knock-in animals *Bra*:EGFP and *MA2*:EGFP knock-in lampreys, respectively). In the lamprey, a T-box family transcription factor *Bra* is expressed in the dorsal protostome (axial mesoderm progenitor), the notochord, and the tailbud^16,17^, while *MA2* is a muscle marker^18–20^. We tested two sgRNAs for *Bra* and one for *MA2* (Table S2). Each sgRNA was co-injected with sgRNA for Tbait (sgT), the donor plasmids, and Cas9 mRNA into zygotes. We incubated the injected embryos and investigated their EGFP expressions during development (Figs. 2 and 3). In this investigation procedure, we screened and counted embryos or prolarvae with specific EGFP expression at stage 16 for *Bra*:EGFP and stage 26 for *MA2*:EGFP knock-in lampreys (Table S3).

With *Bra*-sg1, we observed axial mesoderm progenitor-specific EGFP expression (see Fig. 2A as an example) in 34% (37 of 108) of the surviving animals (Table S3). The remaining 66% showed either sparse, widespread non-specific EGFP expression or no expression. The efficiency was not improved by injecting Cas9 protein instead of mRNA (Table S4). To determine the injection damage and toxicity, we also performed sgRNA1-removed cocktail injection and water injection, comparing the result with the no-injection control. The water injection control showed slightly higher survival rate (28.5%) than that of the sgRNA1-removed cocktail injection control (18.5%), which was within the range of the experimental group (21.0%) (Table S4). As the survival rate of the no injection control was far higher (90.5%) than water injection control, both injection damage and RNA toxicity affect to the survival rate.

At stage 16 (late gastrula), EGFP expression was observed in axial mesoderm-progenitor cells (Fig. 2A) and it was persistently observed in those cells at stage 21 (late neurula) (Fig. 2B). The expression was confirmed histologically (Fig. S2). At stage 25 (late pharyngula), the EGFP expression was restricted mostly to the tailbud and anal regions with some non-specific signals (by unintegrated plasmids) observed in the yolk (Fig. 2C). Likewise, in the case of *Bra*-sg2, we found specific EGFP expression in 23% (25 of 110) of the surviving animals (Table S3). The expression patterns were identical to those observed in *Bra*-sg1-injected animals (Fig. S3). These expression patterns were consistent with reports based on in situ hybridization analysis^16,17^. However, the specific EGFP expression was observed only on one side of the animal body in all examined embryos/prolarvae, suggesting that the integration of the donor plasmid occurred only in some of the blastomeres, causing mosaicism.

In the case of *MA2*-sg1, we observed muscle-specific EGFP expression in 21% (25 of 119) of the surviving animals (Table S3). The EGFP signals were persistently detected in muscle cells of stage 30 ammocoetes larvae (~ 40 days after fertilization; Fig. 3). In all prolarvae examined, the EGFP expression was again restricted to some cells only on the left or right side of the animal body, as observed in the *Bra*:EGFP knock-in lampreys described above. In the animals that expressed EGFP in somatic muscle cells, mosaicism was found even in the same somite (Fig. 3B). Both EGFP-positive and -negative muscle cells were present in these somites, suggesting heterogeneity of mesodermal progenitor cells in somitogenesis. In the animals that expressed EGFP in the head region, the EGFP signals were detected in pharyngeal derivatives, including the upper lip muscle, ventral labial elevator, buccal constrictor, superficial branchial constrictors, interbranchial muscles, and branchial sphincters (Fig. 3A’; nomenclature based on previous description^21,22^).

These results strongly suggest that CRISPR-Cas9-mediated knock-in occurred in some of the blastomeres in the injected animals. To confirm that the integration of the donor plasmid indeed occurred in the targeted loci of the genome, we performed insertion mapping of the *MA2*:EGFP knock-in lampreys (Figs. S4A and S4B). This indicated that the targeted integrations occurred both in the forward (#1 and #2 in Fig. S4B) and reverse (#3 in Fig. S4B) directions. Further evidence of the targeted integration was obtained by the sequencing the transgene-integrated animals (Fig. S4C).

### Generation and photoconversion of *SoxE3*:Dendra2 knock-in lampreys

To explore the potential versatility of our knock-in strategy, we next generated *SoxE3*-targeted knock-in lampreys using donor plasmids that contained the coding sequence for the photoconvertible fluorescent protein Dendra2^23^. In the lamprey, a Sox family transcription factor *SoxE3* is expressed in neural crest cells (NCCs) and plays key roles in pharyngeal development^24,25^. The migration patterns of NCCs have been investigated from the viewpoint of jaw evolution in cell-labeling experiments using lipophilic tracers, such as DiI^20,26,27^. However, these dyes cannot selectively label NCCs, causing inevitable artifact signals. To overcome this problem, we planned to generate *SoxE3*:Dendra2 knock-in lampreys and to perform cell type-specific lineage tracing by selectively highlighting *SoxE3*-positive NCCs.

**Figure 4:**
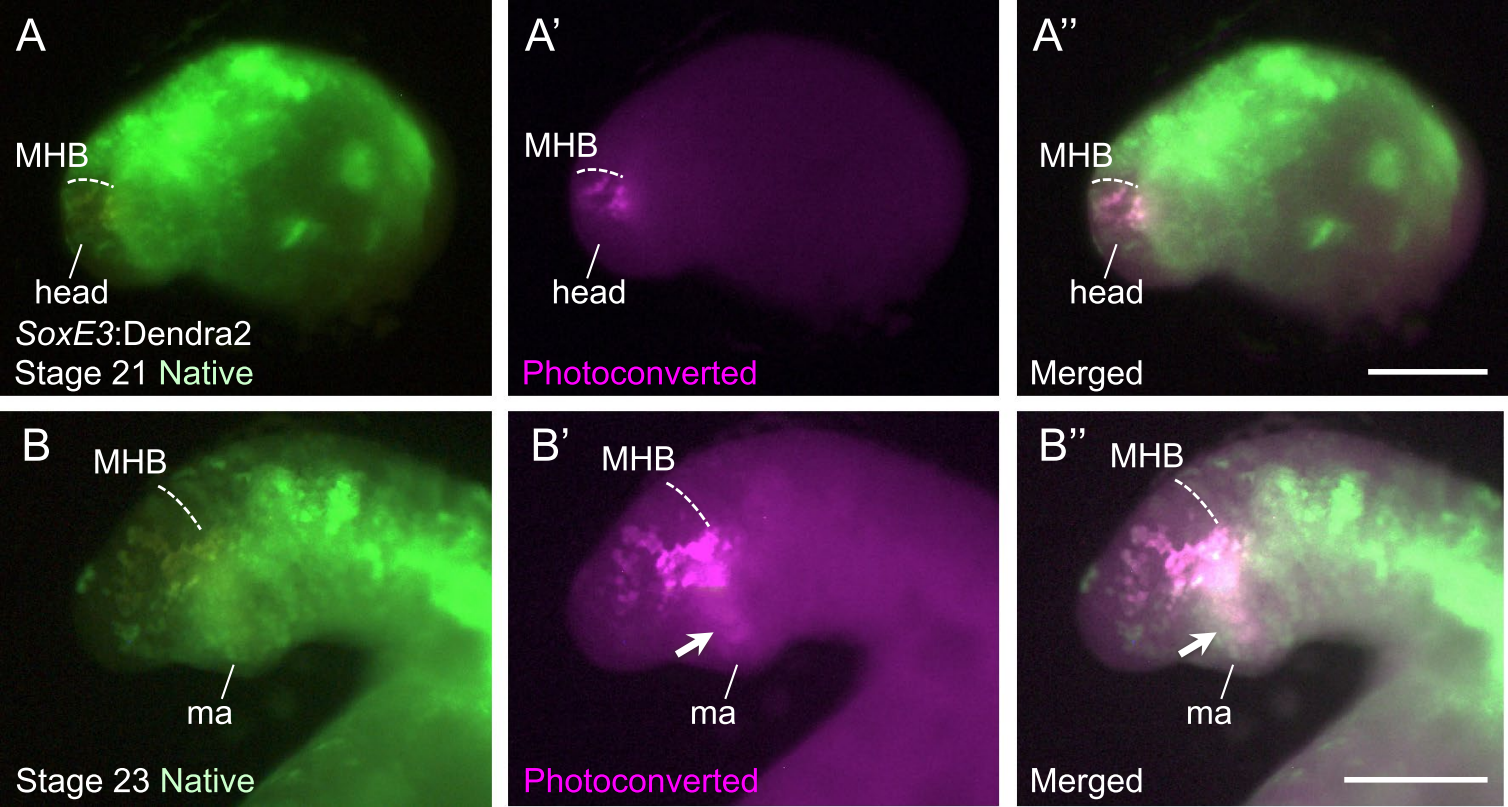
Cell lineage analysis of photoconverted *SoxE3*:Dendra2 knock-in lampreys. (**A**) Photoconversion experiments were performed in the head region of *SoxE3*:Dendra2 knock-in lampreys at stage 21. Native green fluorescence, photoconverted red fluorescence (shown in magenta), and the merged image are shown in (**A**), (**A**’), and (**A**’’), respectively. The midbrain–hindbrain boundary (MHB) is indicated with dashed lines. Scale bar: 500 μm. (**B**) The same animal shown in (**A**) is raised to the stage 24 and reexamined. Some photoconverted cells are observed in the mandibular arch (ma), suggesting ventral migration of these NCC cells (arrows). Native green fluorescence, photoconverted red fluorescence (shown in magenta), and the merged image are shown in (**B**), (**B**’), and (**B**’’), respectively. The midbrain–hindbrain boundary (MHB) is indicated with dashed lines. Scale bar: 200 μm.

For this purpose, sgRNA *SoxE3*-sg1 (Table S2) was co-injected with sgRNA for Tbait (sgT), the donor plasmids containing the Dendra2 sequence, and Cas9 mRNA into zygotes. We incubated the injected embryos and investigated them for their native (green) Dendra2 expression at stage 21. We observed NCC-specific expression in 31% (18 of 59) of the surviving animals (Table S3). We then selectively highlighted some of Dendra2-positive cells by photoconversion with region-specific application with ultraviolet light at this stage (Fig. 4A). We further incubated the photoconversion-treated embryos and re-examined them at stage 24. In these animals, some photoconverted cells (originally situated at the neural tube level on the dorsoventral axis) were observed in the pharyngeal region, suggesting that those cells migrated ventrally (Fig. 4B). These results indicate the usefulness of the CRISPR-Cas9-mediated knock-in system in this kind of lineage tracing analysis.

## Discussion

Although the lamprey is an important organism in evolutionary biology and neuroscience research, no knock-in technique for this animal had not been established. By contrast, knock-in transgenic zebrafish and medaka can be efficiently generated via NHEJ by co-injection of two sgRNAs (one to digest the genome and the other to digest donor plasmids), donor plasmids, and Cas9 mRNA. Here, we showed that this method is perfectly applicable to generate F_0_ knock-in lamprey.

One major impediment in applying modern genetic techniques to the lamprey was that the establishment of transgenic lines is practically impossible due to its long and complex life cycle. This problem would be serious in the situation if we had only low-efficiency genetic engineering techniques. In this study, however, we showed that F_0_ knock-in lampreys can be generated with high efficiency (20–35% of survivors). The higher efficiency compared with zebrafish (around 5%^13^) could possibly result from the slower development of lamprey embryos compared to zebrafish^28,29^. In addition, trained experimenters can perform thousands of microinjections per day^30^. The high efficiency and producibility allow researchers to obtain many knock-in lampreys without the huge effort or time needed to establish transgenic lines. This efficiency was not improved by injecting Cas9 protein (Table S4), although the knock out efficiency approached 99% with co-injection of Cas9 protein and a tyrosinase sgRNA in a previous study^8^. It is possible that the plasmids need to be freshly digested in the cell for efficient knock-in.

Furthermore, sgRNAs design appears to influence the knock-in efficiency. A study reported that the indel-inducing efficiency of each sgRNA correlated with the levels of transient expressions in knock-in zebrafish^13^. As we applied the same method here, we expect that the same correlation will be observed when generating F_0_ knock-in lampreys using this method.

The mosaicism observed broadly in the F_0_ knock-in lampreys imposes an obstacle on the application of this method to some specific experiments. Nevertheless, we have shown that the reporter gene expression in the injected embryos remained until the late developmental period (stage 30 ammocoetes larva, ~ 40 days after fertilization), allowing researchers to perform long-term in vivo experiments in lamprey. This long retention of transgene expression is a major advantage of the CRISPR-Cas9-mediated knock-in method. Furthermore, we have shown that our knock-in system is also applicable to lineage-tracing analysis by generating and photoconverting *SoxE3*:Dendra2 knock-in lampreys. The transgene in the donor plasmid can also be replaced with genes encoding other various proteins such as calcium indicators and channelrhodopsins, enabling researchers to perform calcium imaging, optogenetics, and so on. In our system, the same donor plasmid can be used for different target genes by co-injecting corresponding sgRNAs. The reverse is also true: the same sgRNA for a target gene can be co-injected with different donor plasmids according to various experimental purposes. This flexibility is another significant advantage compared to previous methods such as simple plasmid injection and I-SceI meganuclease-mediated transient transgenesis^11,12,31^. In addition to in vivo imaging and lineage tracing, the method described here has great versatility for various experimental uses in lamprey research.

Finally, we presume that the same strategy can be applied to the vast majority of animals for which one-cell stage embryos are available. As mentioned above, lampreys appear to be particularly amenable to gene editing using the CRISPR/Cas9 system, possibly due to their slow early development time. In addition, the relative abundance of PAM sites due to the high GC content of the lamprey genome^32^ facilitates sgRNA design for genes of interest. Nevertheless, the method established here opens up new research horizons using animals that have previously been regarded as “non-model” organisms in the sense of modern molecular genetics. Although an annotated genome is a de facto requirement for CRISPR/Cas9 genome editing, the present method may conversely facilitate genomic research in “non-model” species.

## Methods

### Ethics approval

This study is reported in accordance with ARRIVE guidelines (https://arriveguidelines.org). All procedures in this study were performed in compliance with the guidelines approved by the animal care and use committees of the National Institutes of Natural Sciences (approved project no. 19A041) and University of Tsukuba (specific approval is not needed for experimentation on fishes at University of Tsukuba, under the Japanese law, Act on Welfare and Management of Animals).

### Animals and embryos

Adult lampreys (*Lethenteron camtschaticum*) were collected from the Shiribeshi-Toshibetsu River, Hokkaido, Japan. In the next spawning season (May to June), the animals were anesthetized in ethyl,3-amino-benzoate methanesulfonate (MS-222). Mature eggs and sperm were squeezed from adults and fertilized in vitro. Embryos and prolarvae were maintained at 16 °C and staged according to Tahara^28^’s description.

### Identification lamprey *heat shock protein 70*-like sequences

A BLASTN search was performed to identify *heat shock protein 70* homologs in a gene model GRAS-LJ^33^ for the *L. camtschaticum* genome assembly LetJap1.0 (BioProject: PRJNA192554) and in transcriptome data obtained from stage 25–26 *L. camtschaticum* larvae^15^ (the raw reads were deposited in the DDBJ Sequence Reads Archives as with an accession number DRA007317), using the zebrafish *hsp70l-1* sequence (ENSDARG00000055723) as a query.

### Phylogenetic analysis of Hsp70 amino acid sequences

For other species, we surveyed Hsp70 sequences from available genomic/transcriptomic databases. The accession/reference numbers for each sequence are shown in Fig. S1. The sequences were aligned using MAFFT^34^ (Katoh and Toh 2008). The best-fitting amino acid substitution model and maximum likelihood (ML) tree were inferred using RAxML software (ver. 8.2.0)^35,36^. The bootstrap values were calculated using 1,000 replicates.

### Isolation of *LcHsp70A* promoter region and construction of donor DNA for knock-in

*L. camtschaticum* genomic DNA was extracted from sperm obtained from a matured *L. camtschaticum* male using DNeasy Blood & Tissue Kit (Qiagen). To amplify *LcHsp70A* promoter region, genomic PCR was performed with the forward primer containing a Not I restriction enzyme recognition sequence and a Tbait sequence (shown in lowercase letters), agagcggccgggctgctgtcagggagctcatggGAAACAAAAGTCGCGCGAGA, and the revers primer containing a BamH I restriction enzyme recognition sequence (shown in lowercase letters), aggatccTGTTACCCCAAACCCTTCGA. The donor plasmid containing Tbait, *LcHsp70A* promoter, and EGFP (Tbait-LcHsP-EGFP) was synthesized from a plasmid described previously^13^, which contains zebrafish heat shock promoter (Tbait-DrHsP-EGFP): Tbait-DrHsP-GFP was digested with Not I and BamH I restriction enzymes and then ligated with the isolated *LcHsp70A* promoter region (Fig. S5). Here, Tbait (GGCTGCTGTCAGGGAGCTCATGG) sequence^13^ was used as a bait sequence in donor plasmids. The plasmid containing Tbait, *LcHsp70A* promoter, and Dendra2 (Tbait-LcHsP-Dendra2) was similarly synthesized from Tbait-LcHsP-EGFP and tetO-Dendra2 plasmid described previously^37^ by BamH I and Xba I restriction enzyme digestion.

### Preparation of sgRNAs and Cas9 mRNAs

The target sites for genome digestion were set approximately 200–600 bp upstream of the predicted transcriptional-start site of the target genes, with the sequence 5′-GA(17 N or 18 N)NGG-3′ or 5′-GG(17 N or 18 N)NGG-3′ with 50–70% GC content. Template DNA for sgRNA synthesize was PCR-amplified from pDR274^38^ with the forward primer, ATTTAGGTGACACTATAgaxxxxxxxxxxxxxxxxxxGTTTTAGAGCTA GAAATAGC (for SP6 polymerase) or TAATACGACT- CACTATAggxxxxxxxxxxxxxxxxxxGTTTTAGAGCTAGAAATAGC (for T7 polymerase), and the reverse primer, AAAAGCACCGACTCGGTGCC. The lowercase letters correspond to genome-targeting sequences in sgRNAs. The genome-targeting sequences in sgRNAs used in this study are shown in Table S2. After PCR amplification with Prime Star Taq polymerase (Takara, Otsu, Japan), PCR product was purified using a QIAquick PCR Purification Kit (Qiagen, Hilden, Germany). Template DNA thus obtained was used for the in vitro transcription of sgRNAs using a MAXIscript T7 kit (Life Technologies, Carlsbad, USA). pCS2-hSpCas9 (gifted from M. Kinoshita and F. Zhang^39^) was digested with NotI, and Cas9 mRNA was transcribed using an mMESSAGEmMACHINE SP6 kit (Life Technologies). sgRNAs and Cas9 mRNA were purified using a RNeasy Mini kit (Qiagen).

### Genomic location of potential off-target sites of sgT

Genomic locations of potential off-target sites of sgT were identified by using the Cas-OFFinder algorithm^40^).

### Microinjection for knock-in

sgRNAs and Cas9 mRNA/protein (Guide-it Recombinant Cas9, 3 µg/µl, Takara Bio USA, Inc., Mountain View, CA, USA) were co-injected into lamprey zygotes with Qiagen miniprep (Qiagen) purified donor DNA in distilled water containing Fast Green to aid visualization of the spread of the injection. Each zygote was injected with ~ 5 nl solution containing ~ 45 pg sgRNA for digesting Tbait, ~ 90 pg sgRNA for digesting genome DNA, ~ 1000 pg Cas9 mRNA/protein, and ~ 45 pg donor plasmid as described previously^14^.

For Microinjection, 2% agar plates were prepared and the agar layer was made holes by a 1000 μl disposal pipette tip, where zygotes were placed. Microinjection was performed under a stereomicroscope, using a microinjector FemtoJet (Eppendolf, Hamburg, Germany) connected to a fine glass pipet placed on a mechanical manipulator.

### Insertion mapping

For insertion mapping, fluorescent F0 knock-in lampreys were collected, and genomic DNA was extracted with standard protocols. The insertion status was examined on either the 5′ side or the 3′ side of the insertion (Fig. S3A). For example, to examine the 5′ side of the insertion, a PCR reaction was performed using a 5′ primer that was specific to each gene (upstream of the expected insertion site) and a 3′ primer that was specific to the donor plasmid (sequence within the *LcHsp70A* promoter for detecting the forward insertion, and sequence within pBluescriptSK for detecting the inverse insertion) as described previously^13^. Some of the amplified fragments were sequenced to examine the joint regions of the insertions.

### Photoconversion

Photoconversion was carried out by illuminating violet-blue light for 30 s using an X-Cite Exacte XCT10A fluorescence microscope illuminater system (Lumen Dynamics, Mississauga, Canada) and a 400–410 nm band-pass filter mounted in a fluorescence microscope BX51WI (Olympus, Tokyo, Japan).

### Histological sections

For histological analysis, specimens were fixed with 4% paraformaldehyde in PBS for 1 h, washed with PBS, and mounted Optimal Cutting Temperature (OCT) compound. Then, the specimens were sectioned using a CM3050 III (Leica) and mounted in SlowFade Diamond Antifade Mountant with DAPI (Thermo Fisher Scientific, Waltham, MA, USA).

### Imaging

Images were taken using a fluorescence stereomicroscope MVX10 (Olympus, Tokyo, Japan).

## Supplementary Information

Supplementary Information.
