# Supplementary material for: Generation of knock-in lampreys by CRISPR-Cas9-mediated genome engineering
Source: Sci Rep. 2021 Oct 6;11:19836. doi: 10.1038/s41598-021-99338-1 (PMC8494898; doi:10.1038/s41598-021-99338-1)
Supplement: Supplementary file 1 — Supplementary Information. [file 41598_2021_99338_MOESM1_ESM.pdf]

Supplementary Information

Generation of knock-in lampreys by CRISPR-Cas9-mediated genome engineering

Daichi G. Suzuki[1, 2], Hiroshi Wada[2], Shin-ichi Higashijima[1, 3]

1. Exploratory Research Center on Life and Living Systems (ExCELLS), National Institutes of Natural Sciences, 5-1 Higashiyama, Myodaiji-cho, Okazaki, Aichi 444-8787, Japan.

2. Faculty of Life and Environmental Sciences, University of Tsukuba, Tsukuba 305-8572, Japan.

3. National Institute for Basic Biology, National Institutes of Natural Sciences, 5-1 Higashiyama, Myodaiji-cho, Okazaki, Aichi 444-8787, Japan.

A

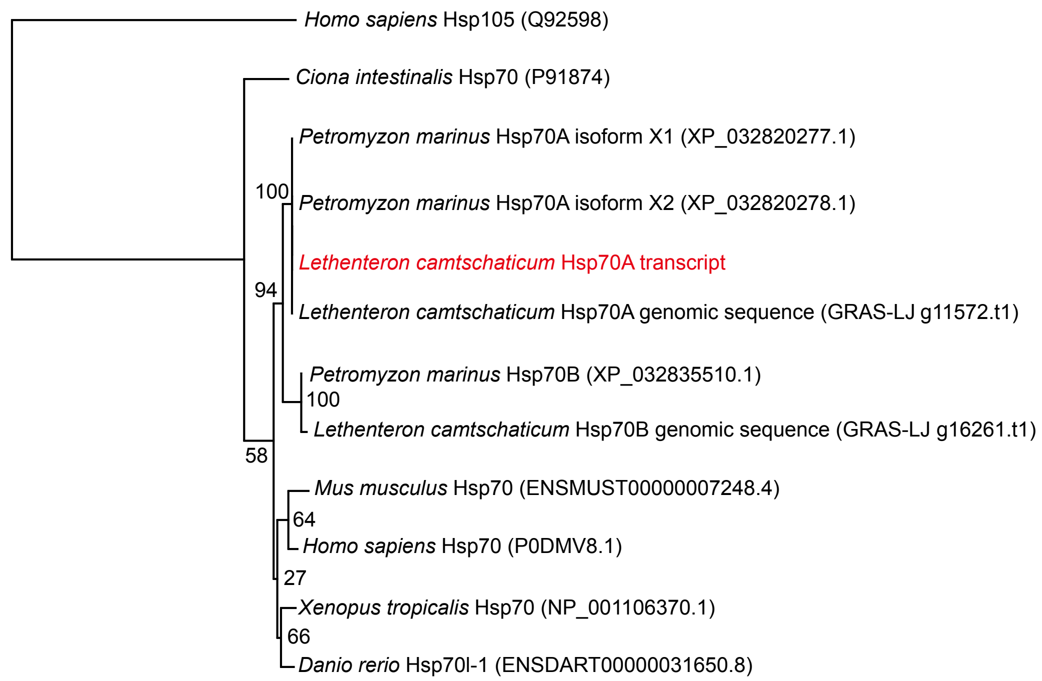

B

...AACGCGGTGGGGGCGGTGGGGGGGTGCGGAAGAGGAAAAAAAATGCGCGCGA**GCCGTGTCCAGGCGGTGCGGCTTCT**

Heat Shock Element (HSE) TATA box

GATTGGCCGCGGGGCGTCACGTGGGCAGAGAATGTTCTGGAAAGCGGGCGGGGTATAAAAGGTGCGCGCAGTGCGGCG

1st exon

TGCGGCCTCACACTCGCGTCCGCCATCAACCGACAGGTCGCGCCGCCCTCTGCCTGCCCCCGAGAACCCTCGACTAAA

AGAAAGGAAAAAAATAAGTACGTGTACGCTTTTATTTCGAAGGGTTTGGGGTAACAAAACTCTGTTTACCTCGCGATA

ATGTTTTAAATTGGTGGTGTTGAGTCGATTGTGGTGTTTTATTTCGCGATTTTCTGTGCCGGGGCCCTCGGTTCGCCG

CCGGCGTCTTCCCGGCTGTGGCTGATGGCGGCCGCGCGGCGTTGGCGGCGGCGTTTATTTAGACTCTCGCTGTGAGGC

GATGTAAGATAATTGTAGTGTTTTTTTTAAG......[ca. 1600 bp]......AAAGAATGACGTATTTCTATGGGTGCAACGTTT

AACGCTTAAAATTAACAATTTTTATTATTTAATATTTTCTTTATGGTTATCGAGATACTTTTTTTAAAATGTGCATGT

2nd exon Start Codon

GTACATGGCAATTCTGACTGATTTTGTTTCCCCCCCCCCTCCTCTCTGGTGCAGACATAAAAATAAAGCAAACATGTC

GAAGGGACCGGCAGTAGGGATCGACCTTGGCACCACCTACTCGTGCGTGGGAGTGTTCCAGCACGGGAAGGTGGAGAT

CATCGCCAACGACCAGGGGAACCGCACCACGCCCTCCTACGTGGCCTTCAATGACTCCGAGCGCCTCATCGGTGATG...

Figure S1. Identification of lamprey heat shock protein 70-like sequences and isolation of *LcHsp70A* promotor region.

(A) Molecular phylogenetic tree for Hsp70 amino acid sequences. The tree was constructed using the ML method. The numbers at the nodes represent bootstrap values.

(B) A partial genomic sequence encoding *LcHsp70A* gene. The sequence isolated as a minimal promotor is shown in bold. Exons and characteristic regions (the heat shock element, TATA box, and start codon) are shown in blue and red, respectively.

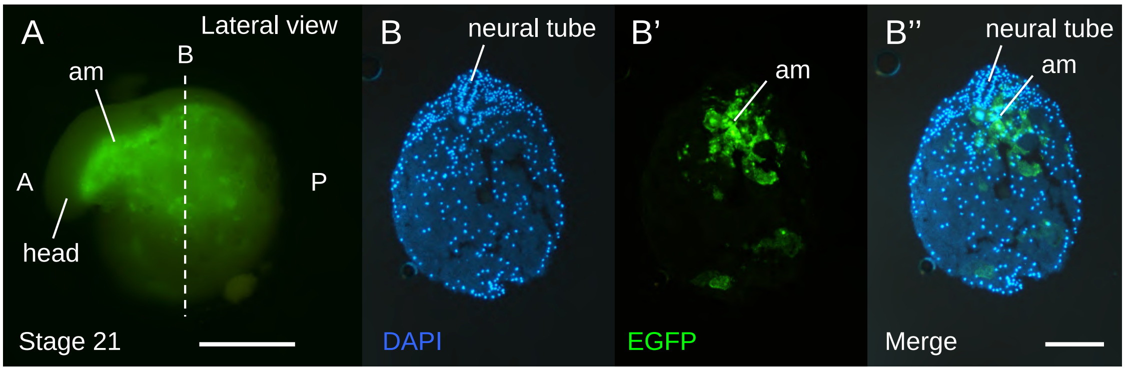

Figure S2. Histological analysis on *Bra*:EGFP transgenic lampreys generated by microinjection of *Bra*-sg1.

(A) At stage 21, in lateral view. EGFP is expressed in the axial mesodermal cells (am). The section plane is indicated by a dashed line. A and P indicate anterior and posterio, respectively. Scale bar: 500 μm. (B) Transverse section of the specimen shown in (A), counterstained with DAPI. Scale bar: 200 μm.

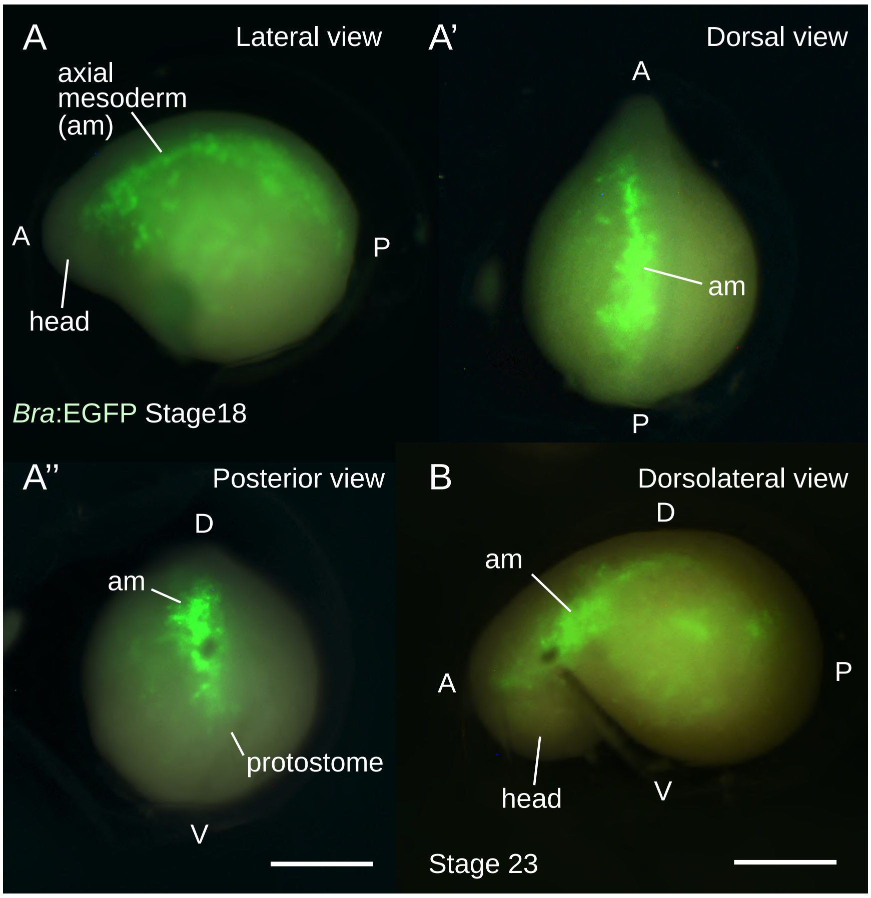

Figure S3. *Bra*:EGFP transgenic lampreys generated by microinjection of *Bra*-sg2.

(A) At stage 18, in lateral view (A), dorsal view (A'), and posterior view (A''). EGFP is expressed in the axial mesodermal cells (am). A, D, P, V indicate anterior, dorsal, posterior, and ventral, respectively. Scale bar: 500 µm. (B) At stage 23, in dorsolateral view. EGFP remains expressed in axial mesodermal cells (am). A, D, P, V indicate anterior, dorsal, posterior, and ventral, respectively. Scale bar: 500 µm.

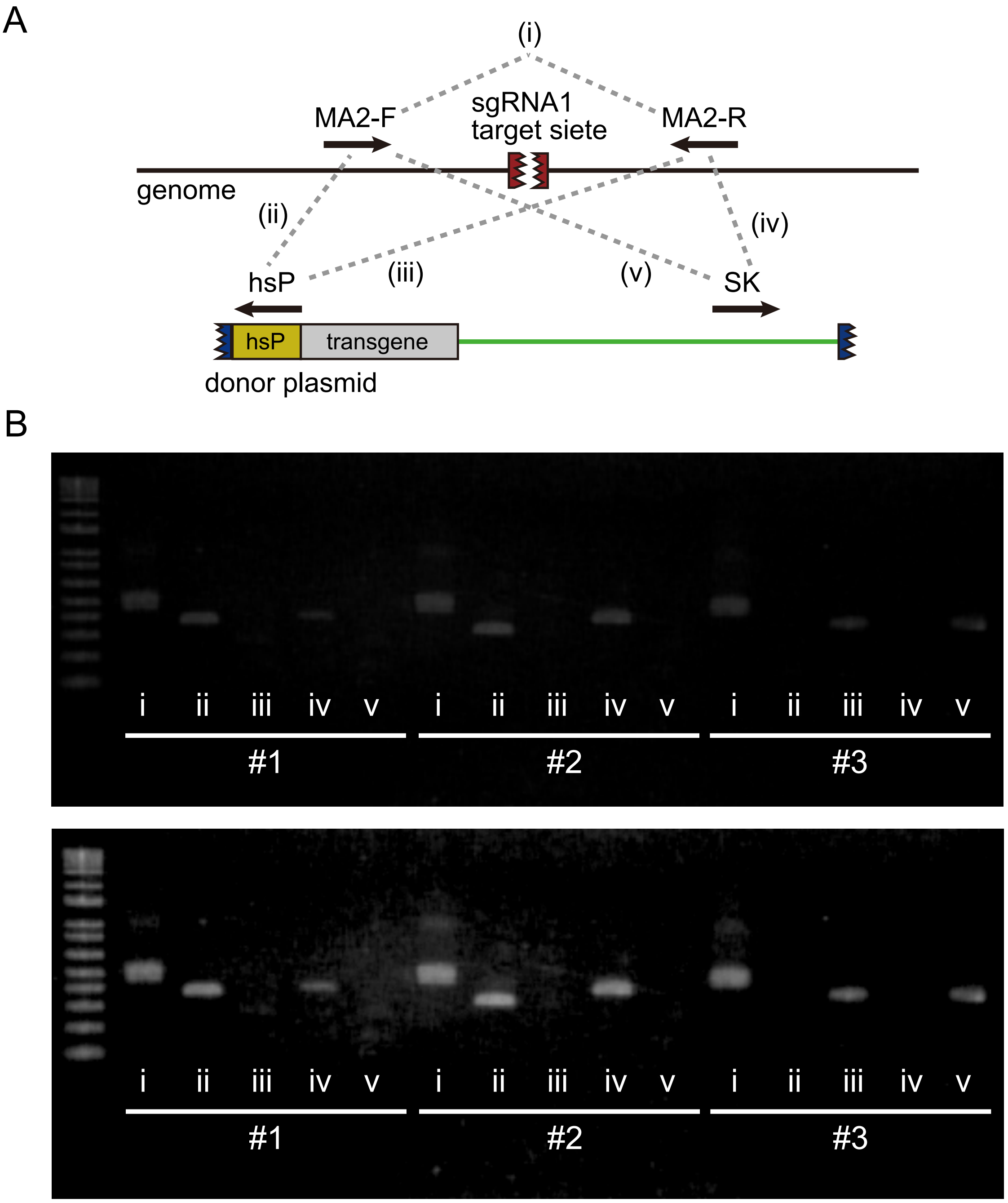

C

5’ side, forward integration

```
                hsP
MA2:EGFP   TGTGGCCCCGAGAAGTCGCGCGAGCCGTGTCCAGGCGGTGCGGCTTCTGATTGGCCG
WT         TGTGGCCCCGAGA--------------------------------------------
```

3’ side, forward integration

```
                Tbait (partial)
MA2:EGFP   GCGGCCGCGGCTGCTGAGGGGGTGTGGGAAGATACAGCTGGGTTTGGGAACGAGGTG
WT         ---------------GAGGGGGTGTGGGAAGATACAGCTGGGTTTGGGAACGAGGTG
```

Figure S4. Insertion mapping and sequence analysis of the *MA2*:EGFP knock-in lampreys.

(A) Primer design for the insertion mapping. Four primers are designed to determine insertion directions in *MA2*:EGFP knock-in lampreys; forward (MA2-F, ATACATAGAACAAGCAAGGGGACCTCT) and reverse (MA2-R, GTTGACCGCCGGGGTCCAGGTTTTATA) primers for the *MA2* coding genomic region, and two primers, for hsP (hsP: TTCGAAATAAAAGCGTACACGTACTTA) and SK (SK: GATTCATTAATGCAGCTGGCACGACAG) regions of the donor plasmid, respectively. Combinations of the primers for genomic PCR analysis are indicated as (i)–(v).

(B) Genomic PCR analysis using each primer combination described in (A). The result for three *MA2*:EGFP knock-in lampreys (#1–#3) are shown. Top: original data. Bottom: contrast adjusted. The blots are not cropped from different parts of the same gel, or from different gels, fields, or exposures.

(C) DNA sequences of the joint region of the insertions for the *MA2*:EGFP knock-in lamprey. The PCR products of the insertion mapping (#1-ii and #1-iv) were sequenced and aligned with the upstream region of the *MA2* gene in the *L. camtschaticum* genome.

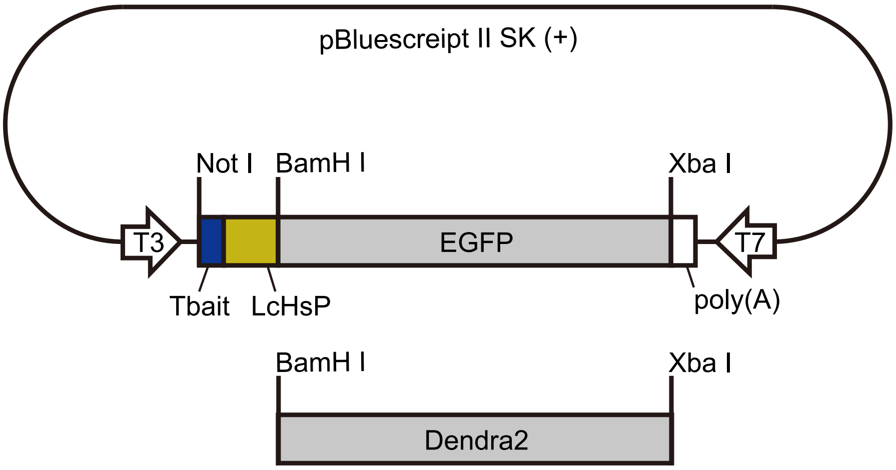

Figure S5. Plasmid design. For Tbait-LcHsP-EGFP) synthesis, pBluescript II SK(+) was used as a backbone. The plasmid contains Tbait, a heat shock promoter region of *L. camtschaticum*, and EGFP. These components are replaceable to other sequences by restriction enzyme digestion and re-ligation.

Accession number	Scaffold or contig number	Position	Sequence	Direction	Number of mismatches
KE993819.1	scaffold00148	988691	cGCTGCTGaCAGGGAGCTCtTGG	-	3
KE993686.1	scaffold00015	1618081	GGCgGCcGTCAaGGcGCTCATGG	+	4
KE993686.1	scaffold00015	2143733	GGtTGCTGTCAGtGtGgTCATGG	-	4
KE993688.1	scaffold00017	1501398	GGCTGCTGTtAGcGAGCgCcTGG	-	4
KE993694.1	scaffold00023	1341836	tGgTGCTGTCAGGGAGCgCAaGG	+	4
KE993720.1	scaffold00049	1997053	GcCTGaTcTCtGGGAGCTCATGG	+	4
KE993729.1	scaffold00058	2250841	aaCTcCTGTCAGGGAGtTCATGG	-	4
KE993730.1	scaffold00059	1303898	GGCTGCTGTtAtcGAGCTCgTGG	+	4
KE993806.1	scaffold00135	430752	tGCTGCTGTCcGGGgGgTCATGG	-	4
KE993823.1	scaffold00152	601079	GGCTGCTGgCAGGGAGCTtcgGG	+	4
KE993860.1	scaffold00189	388288	GGCTGCTGcCAGGGAGCagAgGG	-	4
KE993926.1	scaffold00255	613780	GcCTGCcaTCAGGGAGtTCATGG	+	4
KE993938.1	scaffold00267	638955	GGCTcCTGTagGGGAcCTCATGG	-	4
KE993978.1	scaffold00307	483562	GGtTttTGTaAGGGAGCTCATGG	+	4
KE994039.1	scaffold00368	213088	cGCTGCTGgCAGGGAGgTCAcGG	-	4
KE994043.1	scaffold00372	13980	GGCaGCgGTCtGGGAGCTCtTGG	+	4
KE994099.1	scaffold00428	359739	GGCTGCTGaCAaGGAGgTCAcGG	-	4
KE994105.1	scaffold00434	282568	GGCTGCTGTgAGGacGCTCAaGG	-	4
KE994367.1	scaffold00696	145491	GGCTGCTcTCAGaGAGgTCAcGG	-	4
KE994863.1	scaffold01192	78188	GGCTGCTGTgAGGacGCTCAaGG	+	4
KE995000.1	scaffold01329	10548	GGCaGCgGTCtGGGAGCTCtTGG	+	4
APJL01113700.1	contig086823	1201	GGCaGCgGTCcGGGAGCTCtTGG	-	4
APJL01114005.1	contig087430	2163	tGgTGCTGTCAGGGAGCgCAaGG	+	4
APJL01118947.1	contig112041	1501	GGCaGCgGTCcGGGAGCTCtTGG	-	4
APJL01128773.1	contig112041	726	GGCTGCTGaCAaGGAGgTCAcGG	-	4
APJL01148468.1	contig138789	442	GGCgGCTGaCAtGGAGCaCATGG	-	4
APJL01159461.1	contig152146	505	cGCTGCTGgCAGGGgGCTCAcGG	+	4

Table S1. DNA sequences for sgRNAs against *L. camtschaticum* genomic sequences.

One sequence showed three mismatches and a conserved PAM sequence (5'-NGG) and 26 sequences four mismatches and a conserved PAM sequence compared to the sgT sequence. Mismatches are shown in lowercase letters.

Bra-sg1	GAATTCCGTACGCGGTGGAAGG	(-435 ~ -414)
Bra-sg2	GAGTGGATGTGCCTGTACAGGG	(-468 ~ -446)
MA2-sg1	GGGGGCGGAGAGTTGGTAGTTGG	(-232 ~ -210)
SoxE3-sg1	GGGCGAGGGAGTAAGAGGCGGGG	(-419 ~ -397)

Table S2. DNA sequences for the corresponding sgRNAs.

PAM sequences (5'-NGG) are labeled in red. The numerals shown at the right indicate the locations of the corresponding sequences with respect to the prospective transcription start sites (the 5’end of the longest cDNA) for each gene.

target	reporter gene	sgRNA	check stage	positive	survivor	positive/survivor	dead	total injection
Bra	EGFP	sg1	16	37	108	34.26%	241	349
Bra	EGFP	sg2	16	25	110	22.73%	245	355
MA2	EGFP	sg1	26	25	119	21.01%	361	386
SoxE3	Dendra2	sg1	21	18	59	30.51%	307	366

Table S3. Efficiency of CRISPR-Cas9-mediated knock-in.

	positive	survivor	positive/survivor	dead	survival rate (survivor/total)	total injection
sgRNA1 + sgRNA2 + Cas9 mRNA	11	42	26.2%	158	21.0%	200
sgRNA1 + sgRNA2 + Cas9 protein	7	40	17.5%	160	20.0%	200
sgRNA2 + Cas9 mRNA (no sgRNA1)	N/A	37	N/A	163	18.5%	200
Water only	N/A	57	N/A	143	28.5%	200
No injection	N/A	181	N/A	19	90.5%	200

sgRNA1: *Bra* sg1
N/A: not appliable

Table S4. Control experiments for CRISPR-Cas9-mediated knock-in.
